# Long-term impacts of conservation pasture management in manuresheds on system-level microbiome and antibiotic resistance genes

**DOI:** 10.3389/fmicb.2023.1227006

**Published:** 2023-09-29

**Authors:** Mitiku Mihiret Seyoum, Amanda J. Ashworth, Kristina M. Feye, Steven C. Ricke, Phillip R. Owens, Philip A. Moore, Mary Savin

**Affiliations:** ^1^Department of Crop, Soil, and Environmental Sciences, University of Arkansas, Fayetteville, AR, United States; ^2^USDA-ARS, Poultry Production and Product Safety Research Unit, Fayetteville, AR, United States; ^3^Cellular and Molecular Biology, University of Arkansas, Fayetteville, AR, United States; ^4^Meat Science & Animal Biologics Discovery Program, Department of Animal and Dairy Sciences, University of Wisconsin-Madison, Madison, WI, United States; ^5^USDA-ARS, Dale Bumpers Small Farms Research Center, Booneville, AR, United States

**Keywords:** antibiotic resistance genes, cattle manure, heavy metals, microbial communities, poultry litter, runoff

## Abstract

Animal manure improves soil fertility and organic carbon, but long-term deposition may contribute to antibiotic resistance genes (ARGs) entering the soil-water environment. Additionally, long-term impacts of applying animal manure to soil on the soil-water microbiome, a crucial factor in soil health and fertility, are not well understood. The aim of this study is to assess: (1) impacts of long-term conservation practices on the distribution of ARGs and microbial dynamics in soil, and runoff; and (2) associations between bacterial taxa, heavy metals, soil health indicators, and ARGs in manures, soils, and surface runoff in a study following 15 years of continuous management. This management strategy consists of two conventional and three conservation systems, all receiving annual poultry litter. High throughput sequencing of the 16S ribosomal RNA was carried out on samples of cattle manure, poultry litter, soil, and runoff collected from each manureshed. In addition, four representative ARGs (*intl1*, *sul1*, *ermB,* and *bla_ctx-m-32_*) were quantified from manures, soil, and runoff using quantitative PCR. Results revealed that conventional practice increased soil ARGs, and microbial diversity compared to conservation systems. Further, ARGs were strongly correlated with each other in cattle manure and soil, but not in runoff. After 15-years of conservation practices, relationships existed between heavy metals and ARGs. In the soil, Cu, Fe and Mn were positively linked to *intl1*, *sul1*, and *ermB*, but trends varied in runoff. These findings were further supported by network analyses that indicated complex co-occurrence patterns between bacteria taxa, ARGs, and physicochemical parameters. Overall, this study provides system-level linkages of microbial communities, ARGs, and physicochemical conditions based on long-term conservation practices at the soil-water-animal nexus.

## 1. Introduction

Animal manures are widely used in agriculture as it is a circular strategy for providing macro-and micro-nutrients for soils and crop production. It constitutes an excellent source of nutrients and organic matter (Gurmessa et al., 2021), increases soil carbon levels (Ashworth et al., 2014), enhances soil microbial activity (Ashworth et al., 2017), improves water-holding capacity (Rayne and Aula, 2020), and reduces soil erosion (Heuer et al., 2011; Zhu et al., 2013). However, the consistent surface application of animal manure to soil may also pose potential risks to human health. Studies have shown that manure applications can introduce and disseminate antibiotic residues, antibiotic-resistant bacteria, antibiotic resistance genes (ARGs), and heavy metals to downstream environments (McKinney et al., 2018; He et al., 2020; Zhang et al., 2022). Therefore, to develop effective management and mitigation strategies against the spread of ARGs to the environment, it is crucial to understand their occurrence and distribution in manure-applied systems, as well as their associations with microbial communities and heavy metals.

Manure application, both as deliberate fertilizer treatments and the natural deposition during grazing, likely influences ARGs prevalence in soils, which provides spatially and temporally variable inputs (Hilaire et al., 2022). Studies have shown that this can amplify ARGs levels in the soils and downstream environments (Heuer et al., 2011; Jadeja and Worrich, 2022; Zhang et al., 2022). Specifically, mobile genetic elements can aid in the spread of ARGs through horizontal gene transfer, exacerbating contamination in the environment (Zhang et al., 2017; Vinayamohan et al., 2022). Thus, exchange of ARGs from various environmental bacteria to human and animal pathogenic species poses a real challenge to alleviating global antibiotic resistance (Van Elsas et al., 2003; Forsberg et al., 2014; Nesme and Simonet, 2015; Smalla et al., 2018). This can occur within both the animal gastrointestinal tract (GIT) and in crop production environment, like soil, and can be further facilitated by transferable genetic elements and selective chemical agents (pharmaceuticals, heavy metals and disinfectants) (Frost et al., 2005; Gillings, 2014; Zhao et al., 2019; Wang et al., 2020). Further, in soil fertilized with manure, the longevity of antibiotic-resistant bacteria and their corresponding resistance genes may be extended (Li et al., 2017; Chen et al., 2018; Rahman et al., 2018; Cheng et al., 2019; Yang et al., 2020, 2021). This prolonged survival can be attributed to selective agents that favor their growth, coupled with the extra nutrients the manure provides (Guber et al., 2005; Tien et al., 2017; He et al., 2020; Mware et al., 2022). To date, large numbers of genes conferring resistance to sulfonamide, beta-lactam, macrolide, and tetracycline have been identified in soil that has been fertilized with manure (Peng et al., 2017; Yang et al., 2020).

Antibiotics in animals can select antibiotic-resistant bacteria in the GIT microbiome, which enhances the transfer of ARGs in their manure (Landers et al., 2012; Shterzer and Mizrahi, 2015). Further, antibiotics themselves are not always fully absorbed in the body and thus may be discharged unchanged in the waste, resulting in high antibiotic residuals in the downstream environment (Hilaire et al., 2022). Introduction of animal manure may represent a critical pathway for resistance element introduction into the soil (Yang et al., 2019a,b) (Supplementary Figure S1). Once in the soil, resistance elements can be taken up by plants (Dolliver et al., 2007; Chen et al., 2018; Gao et al., 2018), transported into groundwater or surface water bodies (Dolliver and Gupta, 2008; Song and Guo, 2014; Barrios et al., 2020), or retained in the soil (Cycoń et al., 2019) (Supplementary Figure S1). Studies have shown that ARGs can disseminate from animal farming operations to adjacent agricultural and non-agricultural regions, presenting a substantial risk to long-term environmental health (Wang et al., 2018; Cheng et al., 2019). Given this dissemination, it underscores the critical importance of adopting a One-Health approach, where the interconnectedness of environmental, animal, and human health is recognized and addressed in a holistic manner to effectively combat the spread and impact of antibiotic resistance.

Soil physico-chemical characteristics, including pH, texture, nutrient content, and heavy metal levels, can impact the presence and spread of resistance genes in agricultural soils (Baker-Austin et al., 2006; Rahman et al., 2018; Cycoń et al., 2019; Seyoum et al., 2021a). However, these associations are often complex. For example, the acid content of waste can enhance the antibiotic degradation and reduce corresponding ARGs (Wu et al., 2022). Further, studies analyzing ARGs in various soil types amended with a slurry from swine observed that ARG persistence was negatively linked to organic carbon (Sui et al., 2019; Conde-Cid et al., 2020; Wu et al., 2022). In addition, total nitrogen was also found to be positively linked with *sul*1 gene levels in soils (Sun et al., 2017). Such associations in a long-term manure-applied soil and subsequent water runoff based on management is largely unknown.

Furthermore, despite the crucial role the soil microbiome plays in conservation agriculture, our understanding on the bacterial community’s response and ARG dynamics to sustained manure inputs remains limited, especially in relation to the soil’s physicochemical conditions under ongoing manureshed management. A “manureshed” refers to areas adjacent to animal feeding operations where nutrients from manure can be recycled for agricultural production (Spiegal et al., 2020). Gaining insight into the microbial community dynamics and ARGs within this context can further inform and enhance manureshed-based management practices, promoting better soil health, sustainability, and water quality.

In addition, the effects of cattle manure and poultry litter on the transfer of ARGs through surface transport, and the potential impact of agricultural conservation management practices on the soil and surface runoff microbiome, and the reduction of the spread of ARGs is not well understood. Previous analyses evaluated ARGs and/or microbial communities within specific production environments [i.e., manure (Gurmessa et al., 2021), soil (Yang et al., 2019a,b, 2020), or water (Yang et al., 2021) only]; however, no study has thoroughly evaluated system-level impacts from conservation and conventional practices on ARG dissemination and the manure-soil-water microbiome in long-term.

This research aimed to explore the impact of prolonged use of animal manures (cattle manure and poultry litter) on levels of ARGs, microbial community structures, and environmental parameters in relation to systems-level agricultural conservation management practices over a 15-year period, using a longitudinal study design. Specifically, authors set out to (i) evaluate the impacts of long-term application of animal manure on the distribution of ARGs in the soil and runoff, (ii) assess the microbial community dynamics across the animal manure-soil-runoff system based on long-term conservation (rotationally grazed, rotationally grazed with an unfertilized buffer strip, and rotationally grazed with a fenced unfertilized buffer strip) and conventional (continuously grazed and hayed) management, and (iii) reveal the relationship between the ARGs, bacterial taxa, microbial biomass carbon, and environmental factors in animal manure inputs, soils, and runoff. We predicted that practices such as riparian buffer strips and rotational grazing, which are considered conservation agricultural practices, can reduce the spread of ARGs, and may provide a solution to mitigate the antibiotic resistance associated with manure and/or litter inputs.

## 2. Materials and methods

### 2.1. Description of experimental set-up and treatments

The field setup utilized 15 sloped and individually isolated manuresheds at the USDA-ARS Research Center in Booneville, Arkansas that have been previously described (Pilon et al., 2017a,b, 2019; Anderson et al., 2020; Yang et al., 2021) (Supplementary Figure S2). Three different agricultural conservation management practices were evaluated, including rotationally grazed (R), rotationally grazed with a buffer strip (RB), and rotationally grazed with a fenced riparian buffer (RBR). Two conventional practices were carried out including hayed (H) and continuously grazed (CG). For the H, manuresheds were mowed to 10 cm thrice a year using a rotary hay mower (April, June, and October) without cattle presence. Manuresheds designated as CG underwent year-long grazing, accommodating either one or two calves. R treatments practiced rotational grazing, introducing three steers when forages reached heights between 20 and 25 cm, as measured by a disc meter. These steers were removed when the height was reduced to 10–15 cm.

The RB showcased a 15.3-m buffer strip at their base, uniform in vegetation and spanning an area of 283 m^2^. The RBR manuresheds were characterized by a cattle-excluding fenced riparian buffer zone. This zone was planted in 2003 with a diverse tree assortment: white oak (*Quercus alba* L.), green ash (*Fraxinus pennsylvanica* Marshall), and pecan [*Carya illinoinensis* (Wangenh.) K. Koch], as described in Pilon et al. (2017a).

Each manureshed was divided into three (or four in the RBR treatment) slope positions on the landscape. All manuresheds received poultry litter (excluding the grass buffer strips and riparian buffer strip) and were managed consistently from 2004 to 2019.

### 2.2. Manure sampling and processing

Samples from poultry litter (*n* = 12) were collected at specific timepoints in the spring 2018 and 2019 from in-house piles gathered from a local typical broiler production system. Whereas fresh cattle manure was sampled following typical cattle manure land application practices. For this, 1–2 kg of freshly deposited cattle manure (*n* = 12) was collected from each manureshed undergoing grazing during those springs as described in Gurmessa et al. (2021). The samples were stored in −80°C until further processing. Physico-chemical parameters such as EC, pH, soluble metals, moisture content, total C and N, ammonium-N and nitrate-N were analyzed from both litter and cattle manures as described (Gurmessa et al., 2021).

### 2.3. Soil sampling and processing

From each manuresheds, samples (*n* = 120) were collected from topsoil (0–15 cm) as previously described (Yang et al., 2019a,b, 2020). Samples from each zone (Supplementary Figure S2) were processed and stored in −80°C until further processing. The physico-chemical parameters were evaluated as previously described (Yang et al., 2019a,b; Amorim et al., 2020; Xu et al., 2021; Ashworth et al., 2022). Soil pH was determined with a pH electrode and conductivity meter. Nutrients and heavy metals (Supplementary Table S3) were measured with ICP (I61E Trace analyzer, Thermo Fisher Scientific, Waltham, MA). Total nitrogen (TN) and organic carbon (TOC) were analyzed using the catalyzed high-temperature combustion method (Amorim et al., 2020), and total soil organic carbon (SOC), microbial biomass carbon (MBC) and permanganate oxidizable carbon (POXC) were measured following the procedures in Xu et al. (2021).

### 2.4. Surface runoff sampling and processing

Sampling, filtration, and processing of 100 mL samples from every 95 litter of surface runoff water (*n* = 60) were conducted in 2018 and 2019 as previously described (Yang et al., 2021). To prepare for DNA extraction and subsequent analyses, each runoff sample from the manuresheds was passed through a filtration system. Initially, a sterile membrane filter (45 mm, 0.45-μm pore size, polycarbonate) was positioned on the filter base with its grid side upwards. This was then followed by placing a secondary filter (47 mm, 1.2 μm, cellulose) on top of the initial polycarbonate filter.

### 2.5. DNA extraction, qPCR, and sequencing

DNA was obtained from 0.5 g of poultry litter, cattle manure, and soil (wet weight) as well as runoff water (filter) using MpBio FastDNA Spin extraction kit (MpBio Laboratories, SKU 116560200-*CF*) following manufacturer’s procedures and used for downstream analysis.

Copy numbers of three representative ARGs (*ermB*, *sulI*, and *bla_ctx-m-32_*) and Class 1 integron (*intl1*), were tracked by quantitative PCR (qPCR) from cattle manure, poultry litter, manure applied soil and surface runoff water samples. These ARGs were targeted because they encodes for antibiotics categorized as “Critically Important” for human health according to world health organization reports (WHO, 2014). The standards, primers, amplifications and thermocycling conditions were performed as previously described (Yang et al., 2020, 2021; Gurmessa et al., 2021).

To study microbial communities in manures, soils and surface runoff, the samples were sequenced using Illumina MiSeq sequencing facility targeting the 16S rRNA gene amplicons. The extracted DNA was sent to the Genomic Services Laboratory at the University of Tennessee, where the amplification of the V4 region of the 16S rRNA gene was conducted using barcoded primers 515F and 806R using protocols and conditions described in Yang et al. (2021).

### 2.6. Bioinformatics analysis

Sequences were processed using Qiime2 (v.2021.2) (Bolyen et al., 2019). The analyses code used for this work is available in GitHub repository as Jupyter notebooks (https://github.com/MitikuSeyoum). PCR primers and adaptors were trimmed using Cutadapt (Martin, 2011). The reads were trimmed at 267 and 238 bp for the forward and reverse reads respectively, using the method in the DADA2 (Callahan et al., 2016). Additionally, the initial two bases were removed from both reads.

The sequence reads underwent a process of quality filtering, correction, merging, and removal of chimeric sequences. Then, amplicon sequence variant (ASVs) at 100% sequence identity were generated. Assigning taxonomy was conducted using Naive Bayesian classifier which was trained using the SILVA 138 database (Quast et al., 2013) for bacterial classification.

Bacterial ASVs identified as chloroplast or mitochondrial or non-bacterial, and sequence reads that are unassigned were also removed. A phylogenetic tree for ASV was constructed using the q2-phylogeny plugin, which employed MAFFT 7.3 for sequence alignment and FastTree 2.1 with default settings, as described in Katoh and Standley (2013) and Price et al. (2010) respectively. Measures of alpha diversity such as the number of ASVs, Faith’s phylogenetic diversity, Pielou’s evenness, Shannon’s diversity were calculated as described (Pielou, 1966; Faith, 1992). In addition, beta diversity was estimated based on Bray-Curtis distance (Lozupone et al., 2011) matrices. The beta-diversity distance was ordinated using principal-coordinate analysis (PCoA) and a biplot analysis was conducted to identify the taxa that play a major role in explaining the beta diversity (Halko et al., 2011).

### 2.7. Co-occurrence network analysis

Co-occurrence network was used to uncover the potential interactions between bacteria and ARGs, heavy metals, and microbial biomass carbon in manure, soil, and runoff. A correlation matrix was constructed using all possible pairwise Spearman correlation coefficients (*ρ*) among bacterial taxa and ARGs abundance data obtained from the targeted amplicon analysis and qPCR, respectively. Only statistically significant links with *p* values <0.05 were selected for the network visualizations. Finally, the correlation networks were visualized using Cytoscape (v3.8.2).

### 2.8. Data analysis

Data analyses and graphical visualizations were performed using R (R Core Team, 2020). To determine statistically significant differences, group comparisons were evaluated using non-parametric tests such as the Wilcoxon rank sum test and the Kruskal-Walli’s test. Additionally, spearman’s rank correlation test was also performed to illustrate the link between physico-chemical properties, ARGs and metals and in samples from cattle manure, poultry litter, soil, and runoff water.

Distance metrics were exported from QIIME2 and imported into R to be visualized in a PCoA plot using the available package qiime2R (Bolyen et al., 2019). The Kruskal-Wallis’s test evaluated significant differences in alpha diversity between samples. To visualize microbial community compositions shifts across treatments, PCoA with Bray–Curtis dissimilarity matrices were performed. Pairwise comparisons of beta diversity of microbial community composition between manures, different treatments of soil and surface runoff, and their interactions were performed using PERMANOVA with 999 permutations. When necessary, *p* values were corrected using the Benjamini-Hochberg procedure (Benjamini and Hochberg, 1995) and are described as “q values” in the text.

## 3. Results

### 3.1. ARGs and *intl1* in the manure, soil, and runoff

**Figure 1:**
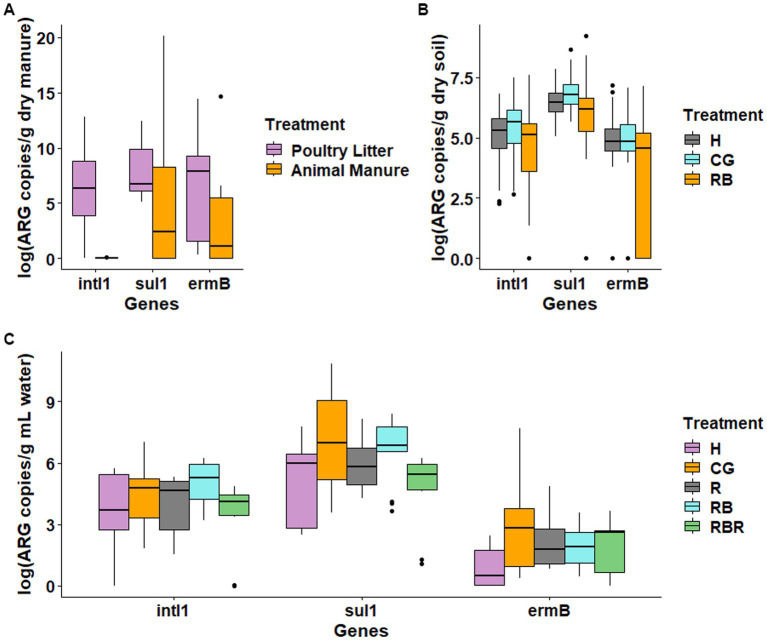
Levels of antibiotic resistance genes in the manures **(A)**, soil **(B)**, and surface runoff water **(C)** samples. RB – rotationally grazed with riparian buffer; R – rotationally grazed; RBR – rotationally grazed with fenced riparian buffer; H – hayed; CG – continuously grazed.

Across the years, ARGs differed in both cattle manure and poultry litter. Specifically, abundance of both ARGs (*ermB* and *sulI*) and *intl1* gene was higher in cattle manure compared to poultry litter (Figure 1A). Among the targeted ARGs, *bla_ctx-m-32_* was below the quantification limits and thus not considered in the data analysis. Among management practices tested, 15 years of continuous grazing resulted in the highest levels *sul1* and *intl1* in the soil (Figure 1B). The monitored ARGs concentration in conservation practices showed reductions (one order of magnitude) compared to conventional practices (Figure 1B). Particularly, differences were observed in both *intl1* (Kruskal-Wallis, H = 6.88, *p* = 0.03) and *sul1* (Kruskal-Wallis, H = 13.032, *p* = 0.0015). Moreover, the levels of *intl1*and *sul1* were 2-log units higher than *ermB*. The absolute abundance of *ermB* was not different (Kruskal-Wallis, H = 5.06, *p* = 0.08) across management practices (Figure 1B).

Similar to manure sources and soil, ARGs such as *sul1* and *ermB*, and *intl1* were consistently found in all runoff water samples, while *bla_ctx-m-32_* was below the limit of detection (Figure 1C). Additionally, among the targeted ARGs, *ermB* was always less concentrated (2-log units lower). Furthermore, there was no difference between pasture management treatments in the quantification of *intl1*, *sul1*, or *ermB* from runoff samples (Figure 1C).

### 3.2. Microbial community structures in manure, soil, and runoff

**Figure 2:**
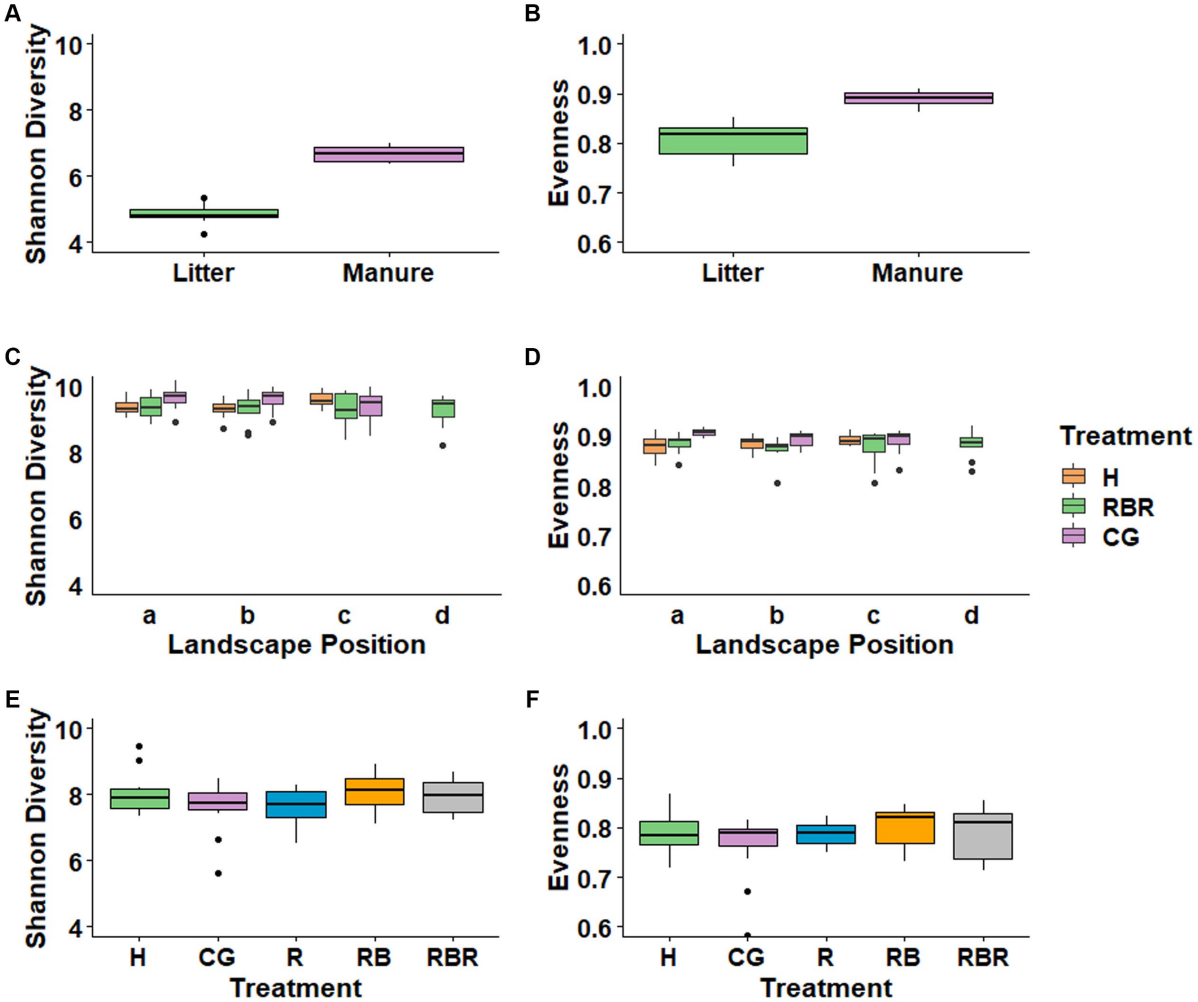
Alpha diversity measures of bacterial communities in different treatments. Evenness and Shannon indices were evaluated in manures **(A,B)**, soil **(C,D)** and surface runoff water **(E,F)**.

To evaluate changes in alpha diversity based on long-term management differences in manuresheds, we calculated total observed ASV, Shannon’s diversity, Pielou’s evenness, and Faith’s PD indices per sample. Results showed that all alpha diversity measures of bacteria increased for cattle manure compared to poultry litter (Figures 2A,B; Supplementary Figure S3). In the soil, alpha diversity measurements of bacteria increased (*p* < 0.05; Kruskal Wallis) in CG compared to H and RG soils in both a and b landscape positions (Figures 2C,D; Supplementary Figure S3). Yet, this trend was not observed in runoff treatments (Figures 2E,F; *p* > 0.05; Kruskal-Wallis).

**Figure 3:**
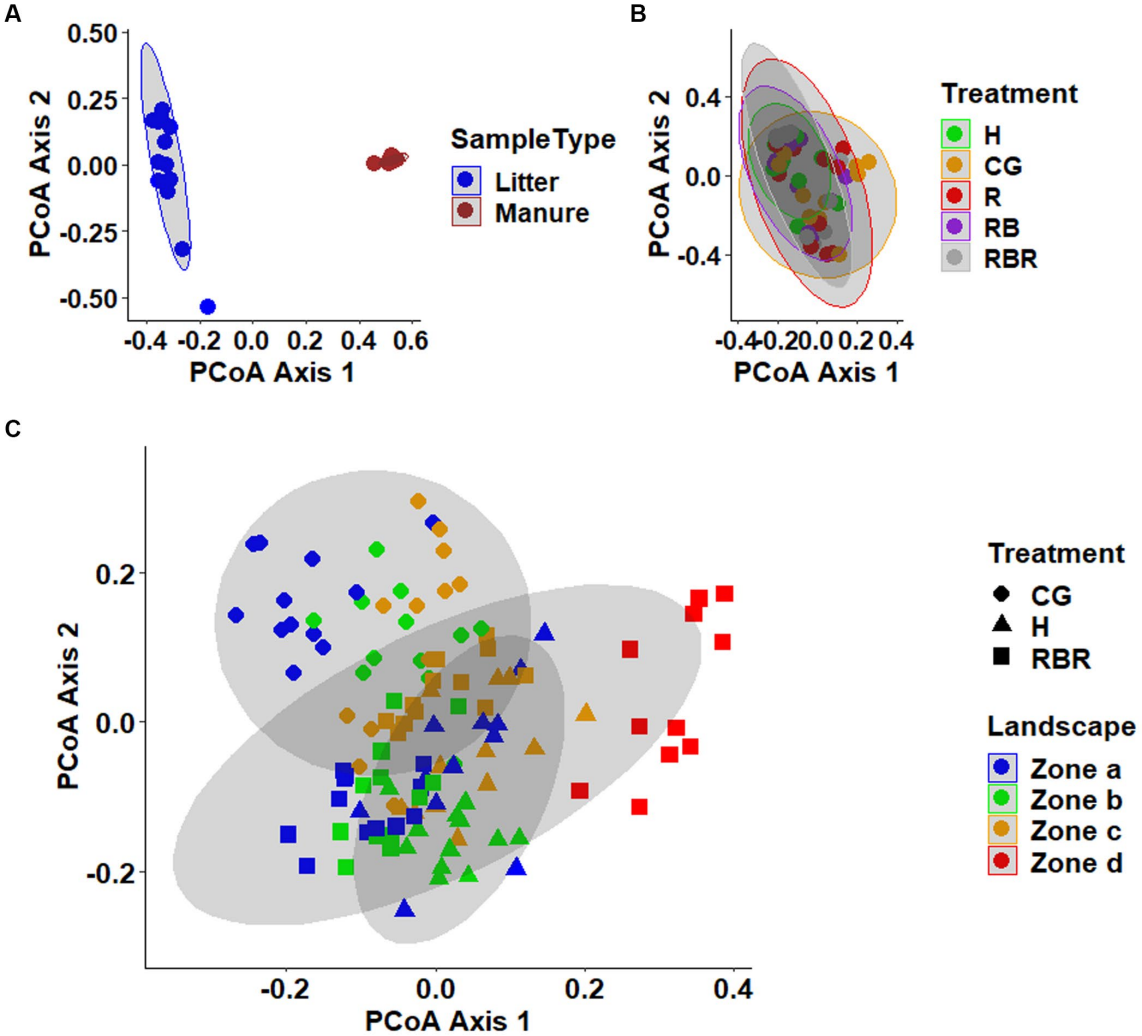
Beta diversity of the manures **(A)**, surface runoff **(B)**, and soil **(C)** microbial communities. Principal coordinates analysis (PCoA) plot based on the Bray-Curtis distance matrix. On the ordination, data points that are closer together are more similar communities.

Analyses of beta diversity in the manures revealed distinct clustering of poultry litter from cattle manure (Pseudo-*F* = 22.97, *p* = 0.001; PERMANOVA), as shown in the PCoA plots (Figure 3A). Notably, in the soil, CG plots showed a separate clustering from conservation practices (Figure 3C). Unlike soil, surface runoff microbial community composition was not changed following agricultural conservation management practices (*p* > 0.05; Figure 3B).

### 3.3. Correlations between bacterial communities, ARGs and environmental factors in manure, soil, and runoff

**Figure 4:**
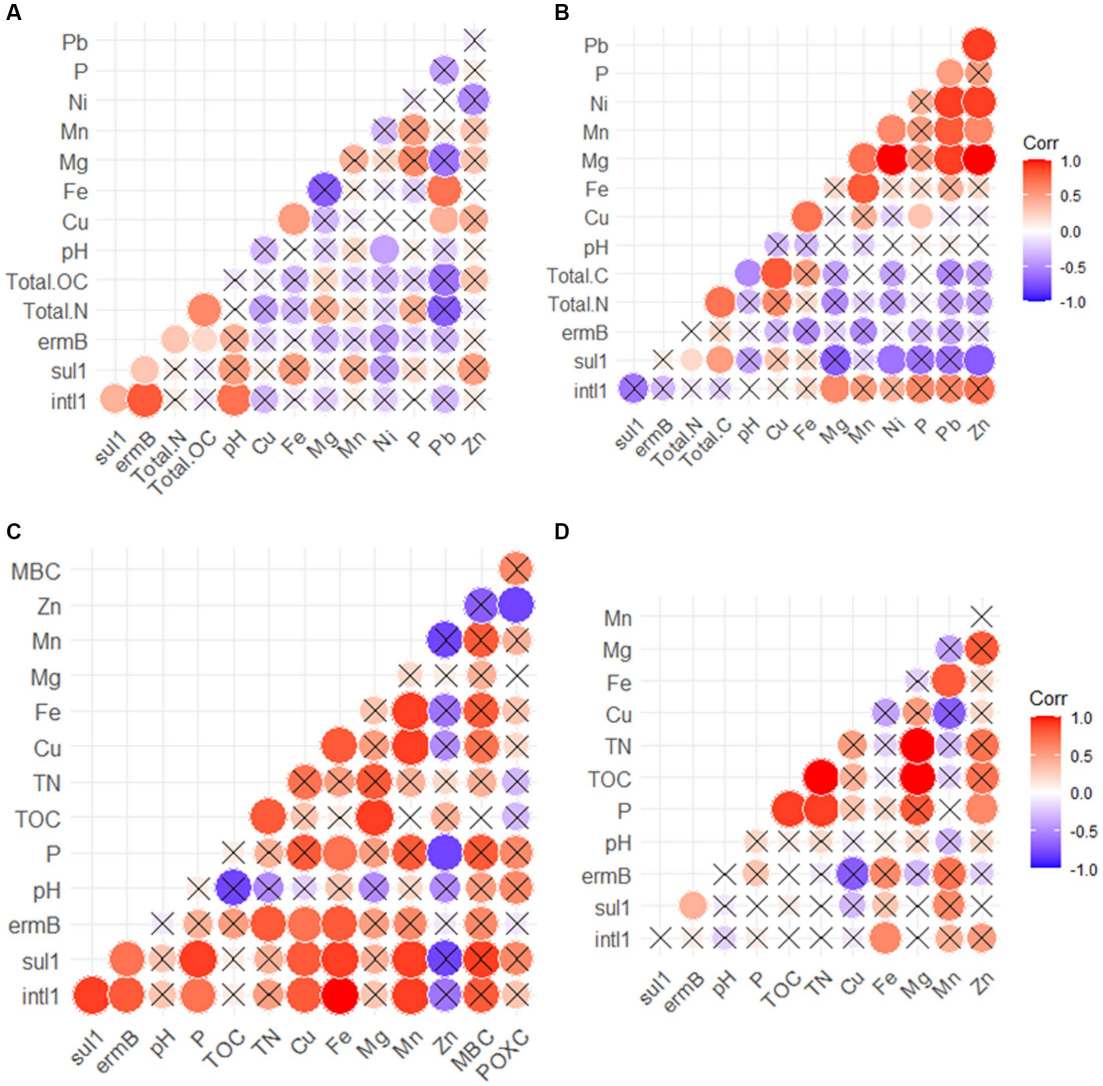
Correlations between heavy metals, levels of ARGs and physicochemical parameters for the cattle manure **(A)**, poultry litter **(B)**, soil **(C)** and runoff **(D)**. Negative relationships are displayed in blue color while positive relationships are displayed in red. The size of the circles and color intensity are proportional to the correlation coefficients. Cross (X) indicates non-significant difference (*p* > 0.05).

In the cattle manure, the *intl1* gene was positively correlated with *sul1*, *ermB* (Supplementary Table S3; Figure 4A), and pH (Figure 4A), but not with any of the metals or nutrients monitored (Figure 4A). Similarly, *sul1* was positively correlated with *ermB* in cattle manure (Supplementary Table S3; Figure 4A). Conversely, none of the detected ARGs were positively correlated with each other or *intl1* in the poultry litter samples (Figure 4B). However, *intl1* showed a positive relationship with Mg (Supplementary Table S3; Figure 4B).

In soil, positive connections were observed between *ermB* and *sul1* (Supplementary Table S3), *sul1* and *intl1* (Supplementary Table S3), as well as *ermB* and *intl1* (Supplementary Table S3; Figure 4C). Similarly, there was a strong correlation between *sul1* and Mn (Supplementary Table S3; Figure 4C), as well as *sul1* and Cu (Supplementary Table S3), *sul1* and Fe (Supplementary Table S3; Figure 4C), and *sul1* and P (Supplementary Table S3; Figure 4C). However, no correlation was found between the detected ARGs and measures of alpha diversity (*p* > 0.05; Figure 4C). Further, spearman analysis revealed that *intl1* was positively linked with Cu (Supplementary Table S3), Fe (Supplementary Table S3), P (Supplementary Table S3), and Mn (Supplementary Table S3; Figure 4C). In addition, MBC, POXC, SOC, and other soil health metrics exhibited non-significant positive associations with ARGs and *intl1* in the soil receiving manure (*p* > 0.05; Figure 4C). In the runoff, inconsistent patterns of relationships were observed compared to soil (Supplementary Table S3). For instance, *sul1* was positively linked with *ermB* (Supplementary Table S3) but not with *intl1* (Supplementary Table S3).

### 3.4. Co-occurrence network analysis in the manure, soil, and runoff

**Figure 5:**
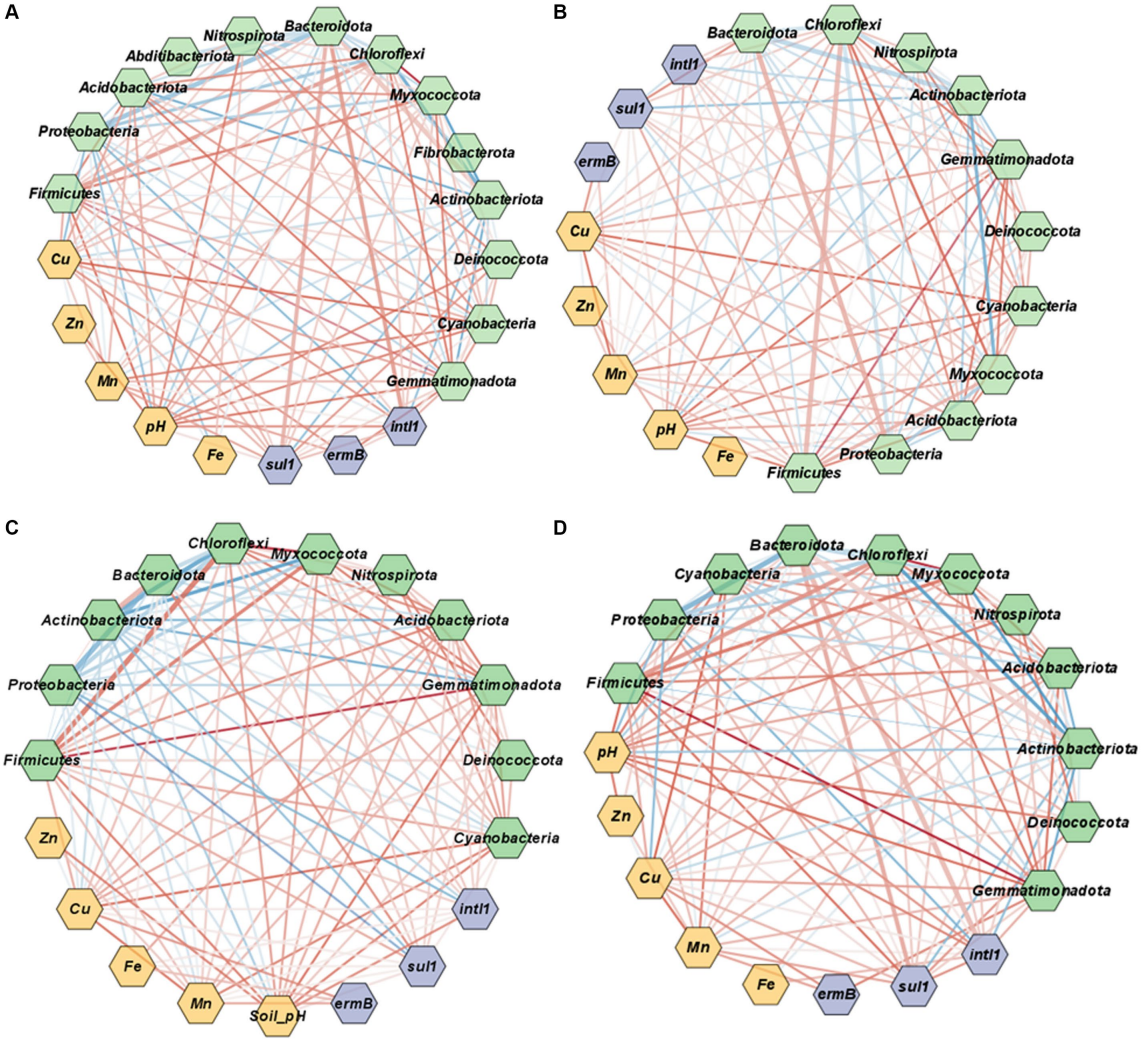
Co-occurrence network associations among ARGs, heavy metals and bacterial taxa at the phylum level in poultry litter **(A)**, cattle manure **(B)**, soil **(C)**, and surface runoff water **(D)**. Purple nodes represent ARGs and *intl1*, light green nodes represent bacterial taxa and orange represent heavy metals and soil properties. The node size is proportional to the connection numbers. The thickness of edge indicates Spearman’s correlation coefficient. Red lines represent positive and significant relationships (*p* < 0.05), and blue lines represent negative and significant relationships between the ARGs, metals and bacterial communities (*p* < 0.05).

The co-occurrence networks representing the associations among ARGs, metals and bacterial taxa were examined to further elucidate potential linkages across properties within the management system for manure, soil, and runoff (Figure 5). Similar to correlation analysis, the co-occurrence analysis demonstrated that both ARGs and heavy metals were positively linked in cattle manure and soil samples (Figures 5A,C), whereas fewer associations were noticed in poultry litter and runoff samples (Figures 5B,D).

In cattle manure, *sul1* showed positive associations with bacterial taxa such as *Bacteroidota* (*ρ* = 0.6, *p* = 0.00), *Proteobacteria* (*ρ* = 0.44, *p* = *0*), *Acidobacteriota* (*ρ* = 0.22, *p* = 0.00). Similarly, *intl1* showed positive associations with *Bacteroidota* (*ρ* = 0.62, *p* = 0.00), *Firmicutes* (*ρ* = 0.55, *p* = 0.00) and *Myxoocooccota* (*ρ* = 0.3, *p* = 0.00).

Notably, *Actinobacteriota* and *Proteobacteria* were negatively correlated with *sul1* in the soil (*p* < 0.01). Further, *Actinobacteriota* has a positive association with *Bacteroidota* (*ρ* = 0.8, *p* = 0.000), but negatively linked with *Firmicutes* (*ρ* = −0.81; Figure 5C) and *Proteobacteria* (*ρ* = −0.45, *p* = 0.00; Figure 5C). Similarly, *Myxoocooccota* had a positive association with *Gemmatimonadota* (*ρ* = 0.3, *p* = 0.00), *Firmicutes* (*ρ* = 0.4, *p* = 0.00), *Acidobacteriota* (*ρ* = 0.83, *p* = 0.00), but negative association with *Bacteroidota* (Spearman, *ρ* = −0.51, *p* = 0.000) and *Actinobacteriota* (Spearman, *ρ* = −0.59, *p* = 0.000; Figure 5C).

In the runoff, *sul1* showed a positive association with *Firmicutes* (*ρ* = 0.75), *Bacteroidota* (*ρ* = 0.62, *p* = 0.000)*, Acidobacteriota* (*ρ* = 0.22, *p* = 0.000; Figure 5D). Similarly, a positive linkage was observed between *intl1* and *Bacteroidota* (*ρ* = 0.6, *p* = 0.000), and *ermB* and *Gammatimonadota* (*ρ* = 0.64, *p* = 0.04; Figure 5D). Furthermore, *Firmicutes* has a positive association with *Chloroflexi* (*ρ* = 0.54, *p* = 0.00), *Gemmatimonadota* (*ρ* = 0.94, *p* = 0.00) and *Acidobacteriota* (*ρ* = 0.67, *p* = 0.00; Figure 5D). Likewise, *Myxococcota* showed a positive linkage with *Firmicutes* (*ρ* = 0.71, *p* = 0.000) and *Gemmatimonadota* (*ρ* = 0.70, *p* = 0.000; Figure 5D) whereas, *Actinobacteriota* had a negative connection with *Chloroflexi* (*ρ* = −0.73, *p* = 0.000) and *Myxococccota* (*ρ* = −0.78, *p* = 0.000; Figure 5D).

## 4. Discussion

Cattle manure and poultry litter contains considerable levels of antibiotic resistant bacteria and ARGs and its applications to soil can enhance the presence of clinically relevant ARGs (Heuer et al., 2011; McKinney et al., 2018; Chi et al., 2022). This study suggests that the increases in the abundance of ARGs and *intl1* in soil that has been continuously grazed may be a result of the direct addition of ARGs and *intl1* via cattle manure as reported by Mware et al. (2022) and Yang et al. (2020), as well as from the indirect enhancement of native soil bacteria that carry ARGs as reported by Macedo et al. (2021). We also observed higher levels of ARGs and *intl1* in cattle manure when compared to poultry litter (Figure 1A), potentially attributable to the administration of antibiotics in cattle farming. Further, the elevated nutrient addition provided by manure inputs, can promote the growth of the soil bacterial community and enrich native bacteria that are resistant to antibiotics (Udikovic-Kolic et al., 2014; Wang et al., 2020). This was also supported by our study where we found a strong association of P with *sul1* and *intl1* in the soil (Figure 4C). In addition, these ARGs are also common in cattle manure (Gurmessa et al., 2021), manure received soils (Chi et al., 2022), and surface water (Yang et al., 2021). Previously, it was reported that levels of *sul1*, *intI1*, *ermB* genes in the soil were greater in overgrazed or continuously grazed pasture fields compared to rotationally grazed pastures, indicating that continuous inputs of manure enhances the levels of ARGs in the soil. Similarly, another study also demonstrated persistence of several ARGs in the soils after 7 years of continuous cattle grazing (Agga et al., 2019).

The proliferation of bacteria introduced via manure, which carry ARGs, can be influenced by the native bacterial community in the soil (Pérez-Valera et al., 2019; Mware et al., 2022). This influence is possibly attributable to competitive interactions (Tien et al., 2017; Barrios et al., 2020). For example, a study found that an initial increase in manure-derived *E. coli* in the soil started to decline, likely due to competition with native bacteria, as well as exposure to UV light and dehydration (Oladeinde et al., 2014; Barrios et al., 2020).

In runoff, there was no significant variation in the prevalence of ARGs between treatments (Figure 1C). There are several possible explanations for differences in the fate of ARGs in runoff. First, ARGs can be taken up by forages in these manuresheds. It is expected that the presence of ARGs and mobile genetic elements reside on the grass of this manuresheds. Consequently, future studies need to include ARG uptake and removal by plants to better understand ARGs fate and uptake pathways in pasture systems. In addition, the levels of ARGs in the surface runoff water can also be impacted by the manure-borne bacteria that can grow and die in the soil. Finally, the majority of manure-associated bacteria may not be available to surface water as most of them remained in the soil zone and thus soil likely became a reservoir that retained significant portion of ARGs.

Strong correlations were identified among ARGs, heavy metals, and physicochemical parameters (Figure 4). We observed positive linkages between *intl1, ermB,* and *sul1* in both cattle manure and soil samples. It is possible that these genes are found together on the same genetic material or within the same host organism, indicating a co-resistance mechanism, as reported by Wang et al. (2019), Wang J. et al. (2022) and Wang Y. et al. (2022). Previously, correlations were also found between *sul1* and *intI1* (Cacace et al., 2019; Seyoum et al., 2021b), which suggests that the spread of *sul1* primarily occurs through lateral gene transfer. Integrons, especially *intl1*, is important genetic elements that plays a vital role in the spread of ARGs (Gillings et al., 2015). Integron genes enable horizontal gene transfer through facilitation of gene addition into other genetic sequences such as plasmids, chromosomes, and transposons (Summers, 2006; Van Hoek et al., 2011; Gillings, 2017).

Co-occurrence network was performed to further understand the linkage patterns of targeted ARGs, heavy metals and bacterial taxa. The microbiome dynamics is considered the key parameter impacting the dissemination of ARGs and the positive linkage between bacterial taxa and ARGs can be predicted as a potential hosts (Awasthi et al., 2019; Deng et al., 2020; Zhu et al., 2021; Chen et al., 2022). In this study, we demonstrated the complexity of associations between tested ARGs and the major bacterial taxa across the manure, soil, and runoff (Figure 5). Such complexity can be important for predicting bacterial species that could be linked with ARGs in different environments. Several previous studies reported microbial taxa as potential ARG hosts (Li et al., 2015; Chen et al., 2019; Wang et al., 2019) and predicted that a strong positive association between the co-existing bacterial taxa and ARGs might provide ARGs related information in the host taxa. In the present study, both *Chloroflexi* and *Bacteroidota* were positively associated with *sul1, intl1,* and *ermB* indicating that these taxa might be suitable potential hosts. In previous reports *Actinobacteria, Firmicutes*, *Bacteroidota*, and *Proteobacteria* were identified to be as the primary hosts associated with ARGs (Peng et al., 2022). Conversely, ARG-carrying bacterial taxa in different environmental matrices may have strong tolerance to environmental stressors (e.g., heavy metals), consequently ARG types carried by them were not affected. For instance, links between microbial community members varied considerably in this study (Figure 5), with some links potentially enabling growth and others inhibiting it. Thus, both *Proteobacteria* and *Actinobacteria* were negatively associated with *sul1* and *intl1*, implying they may function to inhibit proliferation and dissemination of antibiotic-resistant bacteria and their associated ARGs (Zhang et al., 2018; Wang J. et al., 2022; Wang Y. et al., 2022). Several other studies also reported that resistome changes are strongly impacted by microbiomes (Xiang et al., 2018).

In addition, in our study, there were associations between targeted ARGs, heavy metals, and physico-chemical parameters in cattle manure and manured-soil, which is in line with the findings of previous investigations (Zhao et al., 2017; Seyoum et al., 2021a,b). Heavy metals (e.g., Cu, Mn, Zn, and Fe) that accumulate in the soil and runoff can cause co-selection of bacteria, thereby contributing to the dissemination and propagation of ARGs. In our study, Mg showed a positive relationship with *ermB* in runoff. It plays a crucial role in many enzymatic reactions that support microbial metabolism and could potentially affect the relationship with ARGs (Bruna et al., 2022). Furthermore, nutrients in poultry litter and animal manure impacts microbiome, proliferate ARGs-carrying taxa, and affect dissemination ARGs in manured soil and runoff. Previous findings have indicated that ARG levels in animal waste was linked with bacterial community structures (Luo et al., 2017; Shi et al., 2020; Xu et al., 2020; Gurmessa et al., 2021). Previous work also highlighted that composition of bacterial community and gene transfer were primary factors on diversity of ARG in poultry litter, and the poultry environment (Gurmessa et al., 2021; Mazhar et al., 2021), although, the present study identified that poultry litter is not a likely source of ARGs entering the environment (relative to cattle manure).

Overall, conservation pasture systems such as unfertilized edge-of-field grass buffer strips or fenced off riparian buffers can provide a wide range of environmental advantages, such as increased biodiversity, reduction in sediment erosion, and enhanced water quality (Schulte et al., 2016; Anderson et al., 2020; Flater et al., 2022). These buffers were also an effective mitigation strategy for reducing ARGs entering surface waters by an order of magnitude, relative to continuously grazed manuresheds. However, we did not observe any difference in the surface runoff compared to conventional practices. Filter strips mitigate manure-borne microbe and ARG transport and contributes to their use as a best management practice for the important use of manure and poultry litter resources for improving manuresheds’ One Health. Additionally, the concept of One Health acknowledges the interconnectedness of animal, human, and environmental well-being, and requires the cooperation of multiple fields and levels of study to address these issues (McEwen and Collignon, 2018; Banerjee and van der Heijden, 2022). Animal manure introduces organic matter and a diverse array of microbes into soil, directly influencing the quality and safety of both surface and groundwater. Imprudent use of antibiotics in livestock may result in leaching of ARGs into the soil, posing a risk to ground water supplies (Zhang et al., 2021). Manuresheds play a critical role in channeling these ARGs, alongside other contaminants to downstream environments, potentially impacting aquatic ecosystems and human health (Kaiser et al., 2022). Moreover, healthy soils serve as a natural filter, capturing and breaking down many of these potential contaminants. The vitality of the soil microbiome is thus crucial for ensuring both agricultural productivity and the broader ecosystem resilience (Peng et al., 2022). Hence, an integrated One Health strategy that encompasses animal husbandry practices, soil health enhancement, and water quality management is of paramount importance. Recognizing and acting upon these interdependencies can lead to holistic solutions that promote sustainability and health across all three domains.

## 5. Conclusion

This study presents a thorough investigation of system-level linkages of microbial communities, ARGs, heavy metals and physicochemical parameters based on long-term conservation practices at the soil-water-animal nexus. Results demonstrated that conventional systems (continuously grazing) increased soil ARGs compared to conservation practices. Similarly, the diversity and abundance of soil microbial communities increased following long-term continuous grazing compared to conservation management, which was believed to be caused by the consistent addition of manure from animal excrement. Furthermore, there was strong link among heavy metals (Cu, Zn, Fe, and Mn) and targeted ARGs in cattle manure and manured soils, indicating that these elements may play a role in selecting for these genes. However, this linkage was not observed in surface runoff.

While conservation methods, especially the use of unfertilized grass buffer strips and riparian filter strips, have been effective in limiting the spread of ARGs in the soil, the same effect was not demonstrated in runoff. This emphasizes a key observation that conservation efforts lower ARG levels in soil compared to conventional methods, yet the runoff stays largely unchanged. This brings up important questions about how ARGs move and persist in runoff, highlighting a vital area for future research. Understanding this will be essential for developing plans that comprehensively manage the spread of ARGs, focusing on both soil and surface runoff. Furthermore, in line with the One Health perspective, these findings emphasize the need for strategies that consider the interconnected impact on environmental, animal, and human health.

## Data availability statement

The original contributions presented in the study are publicly available. This data can be found here: National Center for Biotechnology Information (NCBI) BioProject, https://www.ncbi.nlm.nih.gov/bioproject/, PRJNA1004307.

## Author contributions

MMS: data analysis, visualization, and writing – original draft. AA: conceptualization, supervision, investigation, methodology, and writing – review and editing. KF and SR: writing – review and editing. PM and PO: investigation, supervision, and writing – review and editing. MS: supervision, writing – review and editing. All authors contributed to the article and approved the submitted version.

## Abbreviations

ARGs: antibiotic resistance genes
CG: continuously grazed
EC: electrical conductivity
qPCR: quantitative polymerase chain reaction
*intlI*: integron integrase 1 gene
*ermB*: erythromycin resistance
ASV: amplicon sequence variant
H: hayed
R: rotationally grazed
LOQ: limit of quantification
MBC: microbial biomass carbon
SOC: soil organic carbon
POXC: permanganate oxidizable carbon.
