# Supplementary material for: Long-term impacts of conservation pasture management in manuresheds on system-level microbiome and antibiotic resistance genes
Source: Front Microbiol. 2023 Sep 29;14:1227006. doi: 10.3389/fmicb.2023.1227006 (PMC10598662; doi:10.3389/fmicb.2023.1227006)
Supplement: Supplementary file 1 [file Data_Sheet_1.docx]

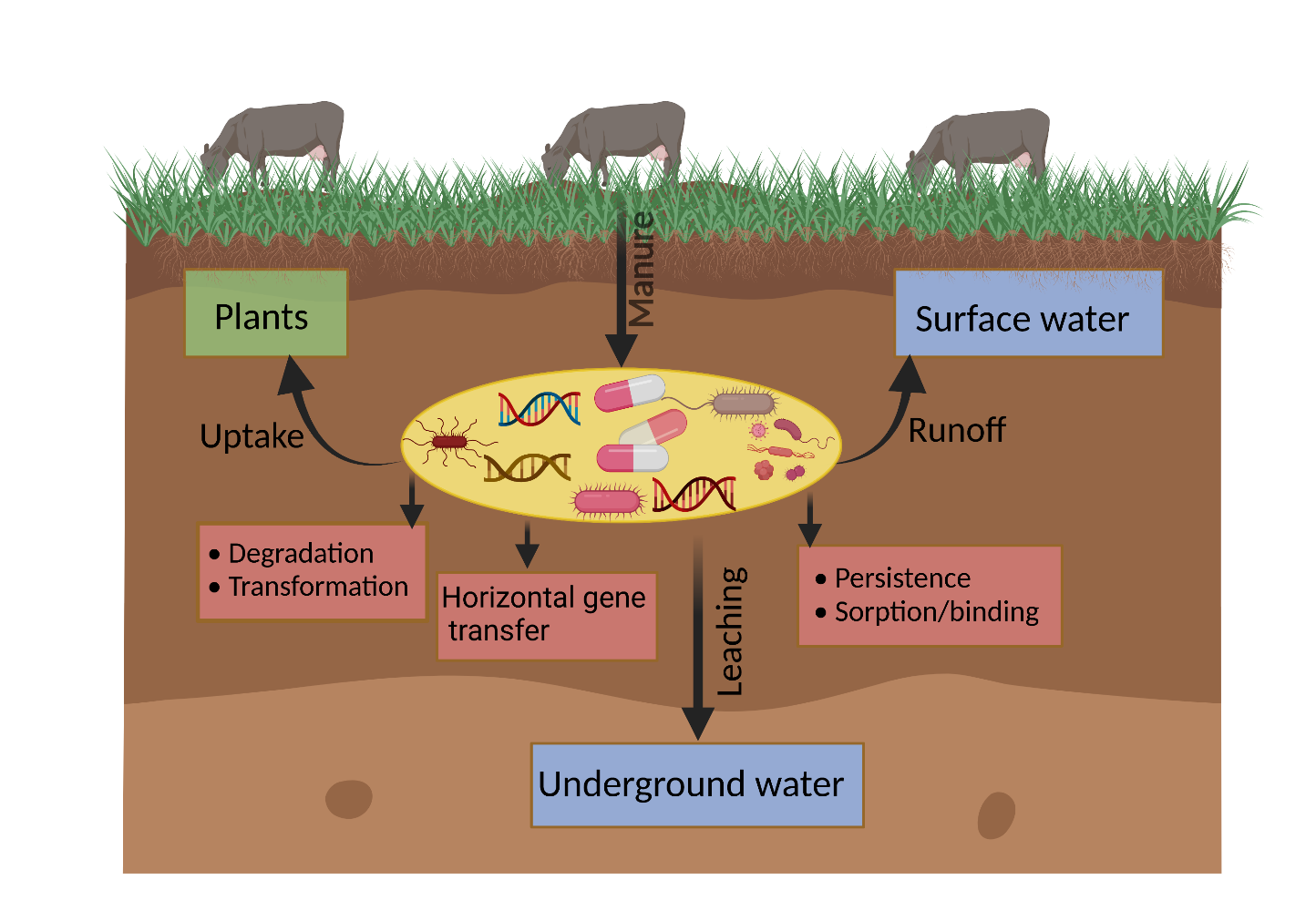


Figure S1: Potential fate of antibiotics and antibiotic resistance genes in the manured soil (Figure created with BioRender)


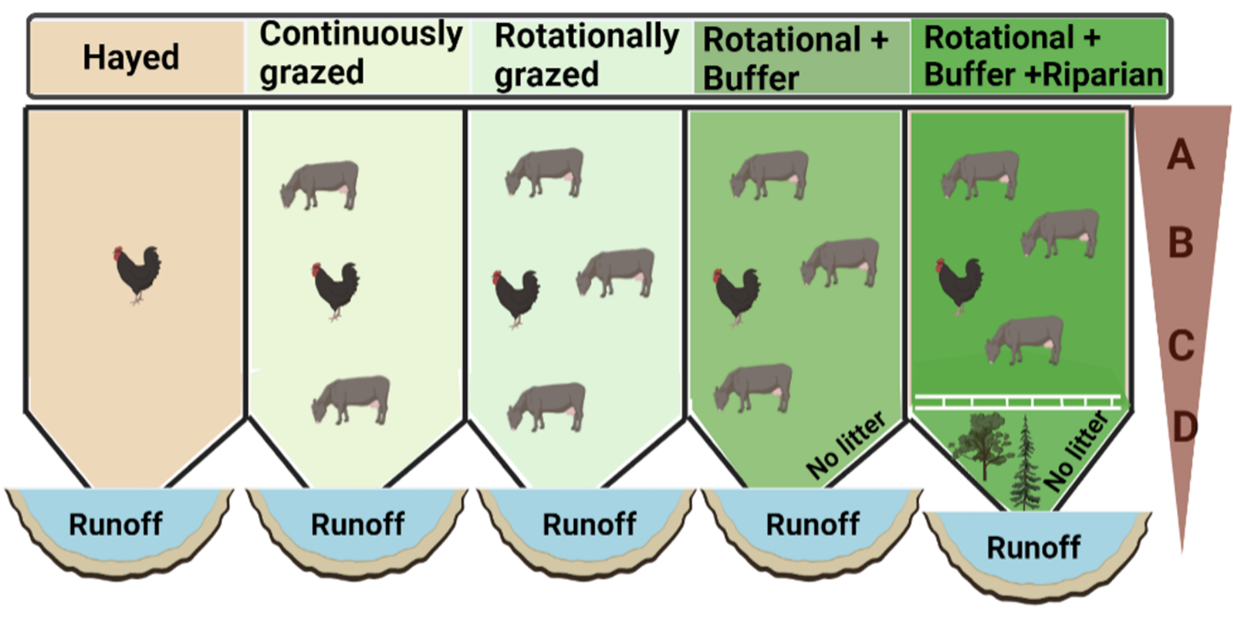


Figure S2: Experimental design setup. Plots were assigned in a randomized complete block design with three replications (15 watersheds total) continuously managed from 2004 to 2019. All areas have received annual poultry litter applications, except at the ‘No litter’ areas indicated. The treatments included: hayed (H), continuously grazed (CG), rotationally grazed (R), rotationally grazed with an unfertilized buffer strip (RB), and rotationally grazed with a fenced riparian buffer (RBR). Each watershed was divided, perpendicular to the slope, into 3 zones [corresponding to shoulder (A), upper backslope (B), and lower backslope positions (C)], whereas RBR had 4 zones (including D).


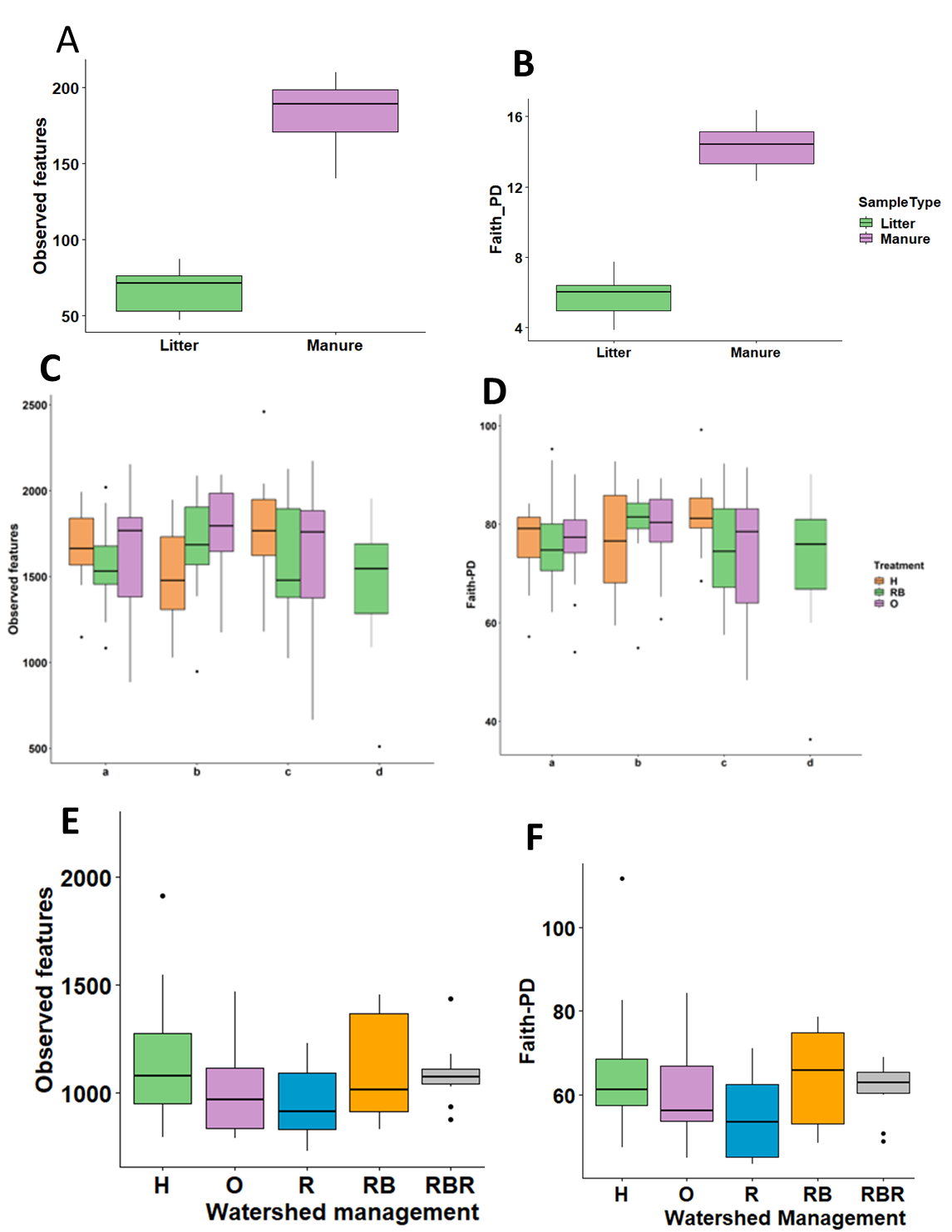


Figure S3: Alpha diversity measures of bacterial communities in Hayed (H), Overgrazed (O) Rotational (R), Rotational grazing with buffer (RB), and Rotational grazing with fenced riparian buffer (RBR) pasture management practices. Observed features and faith-PD were evaluated in Manures (A and B), soil (C and D) and runoff (E and F). Boxes represent 25–75% of the data, solid lines the median, dots in the box mean, the tips represent the minimum and maximum values excluding the outliers (1.5 times lesser or greater than the lower or upper quantiles) represented by dots outside of the boxes.
