# Supplementary material for: Long-term impacts of conservation pasture management in manuresheds on system-level microbiome and antibiotic resistance genes
Source: Front Microbiol. 2023 Sep 29;14:1227006. doi: 10.3389/fmicb.2023.1227006 (PMC10598662; doi:10.3389/fmicb.2023.1227006)
Supplement: Supplementary file 2 [file Data_Sheet_2.docx]

Table S1. Total metal and nutrients contents of cattle manure and poultry litter samples.

| Metal^ǂ^ | Cattle manure, N = 12^1^ | poultry litter, N = 14^1^ | p-value^2^ |
| --- | --- | --- | --- |
| As | <LOQ | 2.3 (1.9) | <0.001 |
| Ca | 20961 (2956) | 29039 (9696) | 0.01 |
| Cd | <LOQ | 0.21 (0.12) | <0.001 |
| Co | 0.26 (0.33) | 2.45 (0.72) | <0.001 |
| Cr | 2.64 (2.18) | 3.38 (1.25) | 0.006 |
| Cu | 61 (23) | 294 (171) | <0.001 |
| Fe | 834 (358) | 727 (505) | 0.7 |
| K | 10925 (4483) | 32927(6349) | <0.001 |
| Mg | 7101 (1679) | 7565 (2656) | 0.7 |
| Mn | 411 (92) | 731(187.71) | <0.001 |
| Mo | 1.96 (0.45) | 3.34 (0.57) | <0.001 |
| Na | 2616 (1435) | 13352 (4960) | <0.001 |
| Ni | 4.5 (1.0) | 15.7 (4.85) | <0.001 |
| P | 12873 (3429) | 17610 (2798) | <0.001 |
| Pb | 0.99 (0.42) | 1.23 (0.58) | 0.087 |
| S | 3573 (938) | 14507 (6226) | <0.001 |
| Se | 0.21 (0.32) | 1.89(0.61) | <0.001 |
| Ti | 2.12 (3.83) | 0.39 (0.31) | 0.2 |
| Zn | 151 (48) | 484.7 (189.2) | <0.001 |

^ǂ^ Measurements were in mg kg^-1^ of dry matter.

<LOQ = below quantification limit

^1^Mean (SD)

^2^Statistical tests performed: Fisher's exact test; Wilcoxon rank-sum test

Table S2. Antibiotics and vaccines used on cattle in grazing treatments from 2004 - 2016 in Booneville, AR.

| **Date** | **# of animals** | **Reason** | **Treatment** | **Amount** |  |
| --- | --- | --- | --- | --- | --- |
| 2004-15 | | 35 | Antibacterial | Covexin 8 | 5 ml each |
| 2004-15 | | 35 | Respiratory | Viralshield 6 | 5 ml each |
| 2004-15 | | 35 | Deworm | Ivomec, Dectomax, or Normectin | 1 ml/33 kg |
| 3/21/16 | | 2 | Ringworm | Fungicide | Topical-amount varies |
| 3/23/16 | | 2 | Ringworm | Fungicide | Topical-amount varies |
| 6/1/16 | | 4 | Pinkeye | Noromycin 300 | 6.6 ml/100 kg |
| 6/1/16 | | 2 | Pinkeye | Noromycin 300 | 6.6 ml/100 kg |
| 6/1/16 | | 32 | Parasites | Cydectin | 1 ml/10 kg |
| 6/3/16 | | 4 | Pinkeye | Noromycin 300 | 6.6 ml/100 kg |
| 6/6/16 | | 4 | Pinkeye | Draxxin | 9 ml each |
| 6/14/16 | | 4 | Pinkeye | Noromycin 300 | 24 ml each |
| 6/20/16 | | 1 | Pinkeye | Duramycin | 46 ml each |
| 6/22/16 | | 1 | Pinkeye | Duramycin | 46 ml each |
| 6/24/16 | | 3 | Pinkeye | Duramycin | 45 ml each |
| 6/27/16 | | 3 | Pinkeye | Liquamycin | 45, 36, 45 ml |
| 6/27/16 | | 3 | Pinkeye | Liquamycin | 45, 36, 36 ml |
| 6/30/16 | | 3 | Pinkeye | Liquamycin | 45, 36, 36 ml |
| 7/5/16 | | 4 | Pinkeye | Penicillin | 1 ml each |
| 7/8/16 | | 4 | Pinkeye | Penicillin | 1 ml each |
| 7/11/16 | | 1 | Pinkeye | Oxytetracycline | 40 ml each |
| 8/31/16 | | 2 | Pinkeye | LA 200 | 40 ml each |
| 9/19/16 | | 5 | Pinkeye | LA 200 | 40 ml each |
| Fall ‘16 | | 35 | Blackleg prevention | 7-way Clostridial | 5 ml each |
| Fall ‘16 | | 35 | Respiratory | Viralshield 6 | 5 ml each |
| Fall ‘16 | | 35 | Pinkeye | Noromycin 300 | 6.6 ml/100 kg |
| 1/25/17 | | 35 | Deworm | SafeGuard | 1 kg/1000 kg body weight |
| 4/18/17 | | 35 | Deworm | Cydectin | 1 ml/10 kg body weight |
| 6/23/17 | | 35 | Deworm/Fly control | Ivermectin | 1 ml/10 kg body weight |

Table S3. Correlation between ARGs and physico-chemicals and heavy metals in poultry litter, cattle manure, and runoff. p-values are added in brackets next to the correlation coefficients (r)

| Variable 1 | Variable 2 | Correlation | | | |
| --- | --- | --- | --- | --- | --- |
|  |  | **Poultry litter** | **Cattle** **manure** | **Soil** | **Runoff** |
| intl1 | sul1 | -0.6 (0.272) | 0.4 (0) | 0.9 (0) | 0 (0.632) |
|  | ermB | -0.3 (0.577) | 0.8 (0.036) | 0.8 (0.026) | 0.1 (0.579) |
|  | Total.N | -0.1 (0.524) | 0.1 (0.47) | - | - |
|  | Total.C | -0.2 (0.475) | -0.1 (0.82) | - | - |
|  | pH | 0 (0.998) | 0.7 (0.033) | 0.3 (0.728) | -0.2 (0.329) |
|  | Cu | 0.1 (0.571) | -0.3 (0.541) | 0.8 (0.017) | -0.1 (0.719) |
|  | Fe | 0.2 (0.785) | -0.1 (0.524) | 1 (0) | 0.6 (0.007) |
|  | Mg | 0.6 (0.035) | -0.2 (0.233) | 0.3 (0.436) | 0 (0.805) |
|  | Mn | 0.5 (0.066) | 0.1 (0.736) | 0.9 (0.011) | 0.4 (0.059) |
|  | Ni | 0.4 (0.11) | -0.2 (0.195) | - | - |
|  | P | 0.6 (0.106) | 0 (0.87) | 0.7 (0.021) | 0.1 (0.493) |
|  | Pb | 0.6 (0.083) | -0.3 (0.867) | - | - |
|  | Zn | 0.7 (0.065) | 0.1 (0.833) | -0.6 (0.129) | 0.5 (0.192) |
|  | MBC | - | - | 0.8 (0.237) | - |
|  | POXC | - | - | 0.3 (0.538) | - |
| sul1 | ermB | 0.1 (0.568) | 0.3 (0.03) | 0.7 (0.028) | 0.4 (0) |
|  | Total.N | 0.2 (0.024) | 0.1 (0.57) | - | - |
|  | Total.C | 0.5 (0.03) | -0.1 (0.558) | - | - |
|  | pH | -0.4 (0.529) | 0.5 (0.13) | 0.3 (0.797) | -0.1 (0.711) |
|  | Cu | 0.3 (0.151) | 0.1 (0.926) | 0.8 (0.012) | -0.3 (0.102) |
|  | Fe | 0.2 (0.171) | 0.5 (0.177) | 0.9 (0.012) | 0.3 (0.249) |
|  | Mg | -0.7 (0.118) | -0.1 (0.202) | 0.4 (0.469) | 0 (0.618) |
|  | Mn | -0.2 (0.502) | 0.4 (0.492) | 0.9 (0.01) | 0.6 (0.083) |
|  | Ni | -0.6 (0.011) | -0.4 (0.261) | - | - |
|  | P | -0.6 (0.439) | 0.2 (0.954) | 0.9 (0.015) | 0 (0.406) |
|  | Pb | -0.6 (0.133) | 0.1 (0.954) | - | - |
|  | Zn | -0.7 (0.018) | 0.5 (0.232) | -0.8 (0.102) | 0 (0.769) |
|  | MBC | - | - | 0.9 (0.327) | - |
|  | POXC | - | - | 0.6 (0.507) | - |
| ermB | Total.N | 0 (0.671) | 0.3 (0.006) | - | - |
|  | Total.C | 0.2 (0.622) | 0.2 (0.013) | - | - |
|  | pH | -0.1 (0.854) | 0.4 (0.578) | -0.1 (0.797) | 0 (0.67) |
|  | Cu | -0.3 (0.639) | -0.2 (0.942) | 0.7 (0.044) | -0.7 (0.069) |
|  | Fe | -0.5 (0.198) | -0.1 (0.879) | 0.8 (0.022) | 0.6 (0.179) |
|  | Mg | -0.2 (0.604) | -0.3 (0.535) | 0.5 (0.189) | -0.3 (0.903) |
|  | Mn | -0.5 (0.357) | -0.2 (0.8) | 0.6 (0.138) | 0.7 (0.057) |
|  | Ni | -0.1 (0.743) | -0.4 (0.324) | - | - |
|  | P | -0.3 (0.671) | -0.2 (0.656) | 0.4 (0.164) | 0.3 (0.345) |
|  | Pb | -0.4 (0.363) | -0.3 (0.523) | - | - |
|  | Zn | -0.2 (0.751) | 0.1 (0.72) | -0.1 (0.593) | -0.2 (0.811) |
|  | MBC | - | - | 0.6 (0.57) | - |
|  | POXC | - | - | -0.1 (0.811) | - |
